# In Vivo and In Vitro Antimicrobial Activity of Biogenic Silver Nanoparticles against *Staphylococcus aureus* Clinical Isolates

**DOI:** 10.3390/ph15020194

**Published:** 2022-02-03

**Authors:** Nashwah G. M. Attallah, Engy Elekhnawy, Walaa A. Negm, Ismail A. Hussein, Fatma Alzahraa Mokhtar, Omnia Momtaz Al-Fakhrany

**Affiliations:** 1 Department of Pharmaceutical Science, College of Pharmacy, Princess Nourah Bint Abdulrahman University, P.O. Box 84428, Riyadh 11671, Saudi Arabia; ngmohamed@pnu.edu.sa; 2 Department of Pharmaceutical Microbiology, Faculty of Pharmacy, Tanta University, Tanta 31527, Egypt; omina.elfakharany@pharm.tanta.edu.eg; 3 Department of Pharmacognosy, Faculty of Pharmacy, Tanta University, Tanta 31527, Egypt; 4 Department of Pharmacognosy and Medicinal Plants, Faculty of Pharmacy (Boys), Al-Azhar University, Cairo 11884, Egypt; ismaila.hussein@azhar.edu.eg; 5 Department of Pharmacognosy, Faculty of Pharmacy, Alsalam University, Tanta 3111, Egypt; drfatmaalzahraa1950@gmail.com

**Keywords:** AgNPs, antioxidant activity, flow cytometry, *Gardenia thailandica*, HPLC, infected wound, qRT-PCR

## Abstract

*Staphylococcus aureus* can cause a wide range of severe infections owing to its multiple virulence factors in addition to its resistance to multiple antimicrobials; therefore, novel antimicrobials are needed. Herein, we used *Gardenia thailandica* leaf extract (GTLE), for the first time for the biogenic synthesis of silver nanoparticles (AgNPs). The active constituents of GTLE were identified by HPLC, including chlorogenic acid (1441.03 μg/g) from phenolic acids, and quercetin-3-rutinoside (2477.37 μg/g) and apigenin-7-glucoside (605.60 μg/g) from flavonoids. In addition, the antioxidant activity of GTLE was evaluated. The synthesized AgNPs were characterized using ultraviolet-visible spectroscopy, Fourier-transform infrared spectroscopy, transmission and scanning electron microscopy (SEM), zeta potential, dynamic light scattering, and X-ray diffraction. The formed AgNPs had a spherical shape with a particle size range of 11.02–17.92 nm. The antimicrobial activity of AgNPs was investigated in vitro and in vivo against *S. aureus* clinical isolates. The minimum inhibitory concentration (MIC) of AgNPs ranged from 4 to 64 µg/mL. AgNPs significantly decreased the membrane integrity of 45.8% of the isolates and reduced the membrane potential by flow cytometry. AgNPs resulted in morphological changes observed by SEM. Furthermore, qRT-PCR was utilized to examine the effect of AgNPs on the gene expression of the efflux pump genes *nor*A, *nor*B, and *nor*C. The in vivo examination was performed on wounds infected with *S. aureus* bacteria in rats. AgNPs resulted in epidermis regeneration and reduction in the infiltration of inflammatory cells. Thus, GTLE could be a vital source for the production of AgNPs, which exhibited promising in vivo and in vitro antibacterial activity against *S. aureus* bacteria.

## 1. Introduction

Nanotechnology is a relatively novel discipline with massive applications, including those in the medical and pharmacological industries [1]. Recently, there is a growing interest in the usage of green-synthesized biocompatible silver nanoparticles (AgNPs) in various applications including antimicrobial products, anti-fungal preparations, drug delivery, the textile industry, and food packaging. Several chemical and physical methods for the synthesis of AgNPs have been reported, including sol-gel, chemical reduction, physical vapor deposition, thermal decomposition, and microwave irradiation [2,3]. Unfortunately, these techniques have many drawbacks such as the expense, use of high energy and/or hazardous chemicals, and production of toxic byproducts that are unsafe to the environment [4,5]. Besides, these toxic chemicals are attached to the end products, which considerably limits their application [2,6].

To overcome these limitations, it is crucial to find eco-friendly, easy to use, cost effective, and nontoxic alternative methods for the fabrication of AgNPs [4]. Recently, new methods based on green synthesis are emerging. These methods use eco-friendly compounds as reducing agents [1]. Plants and microorganisms are considered nontoxic biological reproducible resources that are safe for humans and the environment. They may be more suitable alternatives for the biosynthesis of AgNPs [7]. Plant extract nanoparticles are more favorable than microorganism-based nanoparticles since they do not require particular and complex processes such as culture management, isolation, and several purification steps [8]. In addition, using plants for the synthesis of nanoparticles has other advantages, such as the use of safer solvents, milder response conditions, more feasibility, and their various uses in surgical and pharmaceutical applications [3]. Due to the aforementioned limitations, researchers have developed green methods that employ various plant parts such as the leaf, peel, flower, fruit, and root. Numerous plant extract compounds (e.g., ascorbic acids, flavonoids, polyphenols, proteins, and terpenoids) play important roles in metal ion uptake, precursor salt reduction, and capping agents. Furthermore, several of them have antibacterial capabilities [9].

*Gardenia thailandica* Triveng. is a flowering plant native to Thailand. Gardenia species have a high medicinal potential and a long history of usage in traditional medicine to cure a variety of ailments such as jaundice, fever, hypertension, and skin ulcers. In addition, various Gardenia species have been linked to a variety of pharmacological effects, including anti-inflammatory, anti-viral, anti-cancer, and anti-apoptotic properties [10,11,12,13]. One of the most potential antimicrobials used in nanomedicine are AgNPs. AgNPs can interact with a microorganism’s cell wall, producing reactive oxygen species that eventually cause cell death [9]. As a result, we can speculate that using *G. thailandica* extract will produce AgNPs with improved antimicrobial activity.

*Staphylococcus aureus* is a highly virulent pathogenic bacteria that can cause various clinical infections in humans. They are a major cause of infective endocarditis and bacteremia, in addition to skin and soft tissue, osteoarticular, pleuropulmonary, and device-associated infections [14]. Besides the various virulence factors they possess, antimicrobial resistance is widely spreading among these bacteria. Thus, new approaches should be studied to overcome different infections caused by *S. aureus* [15]. The green synthesized AgNPs could be a therapeutic alternative to the currently present antimicrobials.

In this study, we aimed to green synthesize AgNPs from *Gardenia thailandica* leaf extract (GTLE). Then, the produced AgNPs were characterized by different techniques. Furthermore, the antibacterial activity of the synthesized AgNPs was studied both in vitro and in vivo against *S. aureus* clinical isolates.

## 2. Results

### 2.1. High Performance Liquid Chromatographic Coupled with Diode Array Detector (HPLC-DAD) Analysis

**Figure 1:**
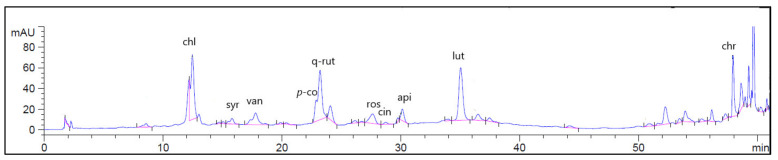
HPLC-DAD of GTLE (320 nm). Chl—chlorogenic acid; syr—syringic acid; van—vanillic acid; *p-*co—*p-*coumaric acid; q-rut—quercetin rutinoside; ros—rosmarinic acid; cin—cinnamic acid; api—apigenin; lut—luteolin; chr—chrysin.

The identification and quantification of phenolic compounds of GTLE was performed using the HPLC-DAD. Figure 1 displays the HPLC-DAD chromatogram for the identified flavonoids and phenolic compounds of GTLE. The abundant phenolic compounds were chlorogenic acid (1441.03 μg/g), while the major identified flavonoid compound was quercetin-3-rutinoside (2477.37 μg/g), as shown in Table 1.

### 2.2. Characterization of the Green-Synthesized AgNPs

#### 2.2.1. Physical Observation

After 3 h of preservation in a cool and dark area, the physical appearance of the AgNO_3_ solution changed to a dark solution after the addition of GTLE, indicating the chemical reduction reaction and synthesis of AgNPs.

#### 2.2.2. UV-Vis Spectroscopy

**Figure 2:**
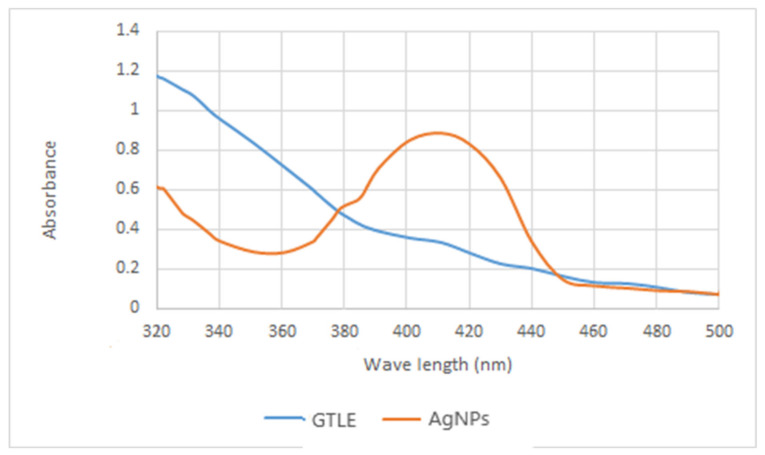
UV spectrum of the biosynthesized AgNPs by GTLE compared to GTLE.

UV-Vis spectroscopy was utilized as the first proof of nanoparticle formation owing to the selectivity of UV towards the formed nanoparticles. Since AgNPs have a characteristic optical reflectivity, they interact strongly with specific wavelengths of light. Because of the collective oscillation of electrons in AgNPs, free electrons produce a surface plasmon resonance (SPR) absorption band [16]. The absorption of AgNPs is controlled by the dielectric medium, chemical environment, shape of the particles, and particle size. The UV measurements of the produced AgNPs had an absorbance at 418 nm (Figure 2).

#### 2.2.3. Fourier-Transform Infrared (FTIR) Spectroscopy

**Figure 3:**
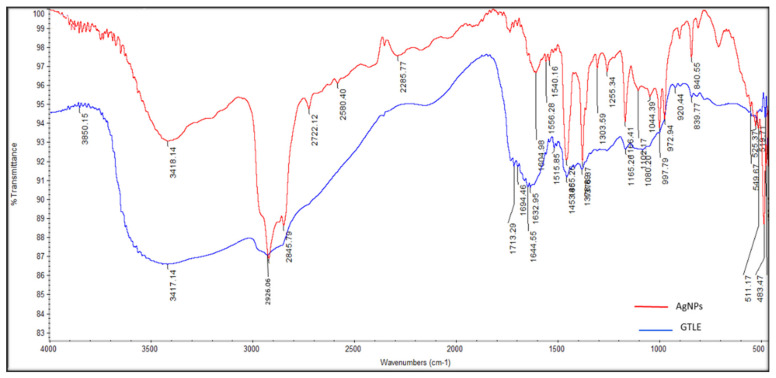
FTIR spectrum of the biosynthesized AgNPs by GTLE compared to GTLE.

The identity of the functional chemical groups of the GTLE involved in the reduction reaction to produce AgNPs was configurated by FTIR spectroscopy measurements. Peaks at 3417, 2926, 1632 cm^−1^ represent the functional groups as follows; OH, C aliphatic, and C=O of phenolic acids and flavonoids, while the polyphenols and aromatic compounds were represented by the peak at 1453 cm^−1^. The secondary OH groups of GTLE were confirmed by the peak at 1080 cm^−1^ (Figure 3).

#### 2.2.4. High-Resolution Transmission Electron Microscope (HR-TEM)

**Figure 4:**
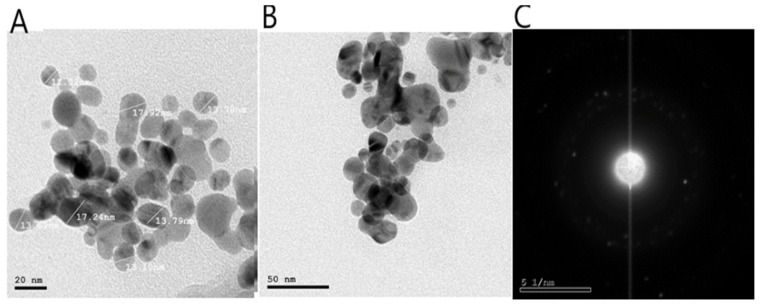
HR-TEM micrographs of the biosynthesized AgNPs using GTLE; (**A**): at 20 nm, (**B**); at 50 nm, (**C**): SAED confirmed the crystalline nature of the formed AgNPs.

The green-synthesized AgNPs using GTLE as a reducing agent were examined using HR-TEM, which revealed the formation of spherical shaped AgNPs with a particle size range of 11.02–17.92 nm and an average size of 14.24 nm (Figure 4). In addition, the selected area electron diffraction (SAED) pattern confirmed the crystalline nature of the formed AgNPs.

#### 2.2.5. Zeta Potential and Dynamic Light Scattering (DLS)

**Figure 5:**
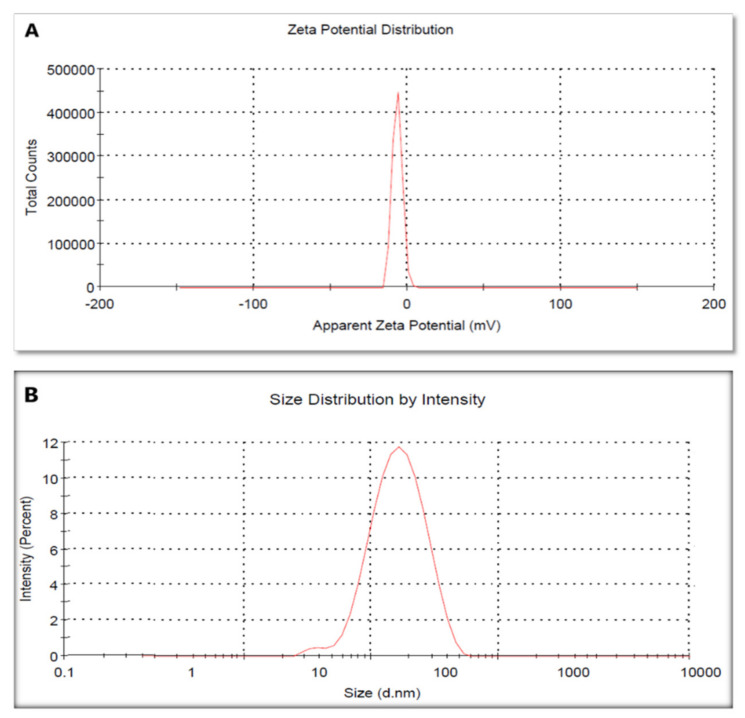
Zeta potential analysis (**A**) and DLS (**B**) of the biosynthesized AgNPs by GTLE.

We used the zeta potential technique to evaluate the surface charge of the green-synthesized AgNPs. Herein, AgNPs had a zeta potential value of −6.54 ± 0.6 mV, where the negative charge highlighted the stability of the formed nanoparticles (Figure 5A).

Legend shells including the metallic shell of the formed nanoparticles were measured using the DLS technique; they had a size of 77.4 ± 1.88 nm (Figure 5B).

#### 2.2.6. X-ray Diffraction (XRD)

**Figure 6:**
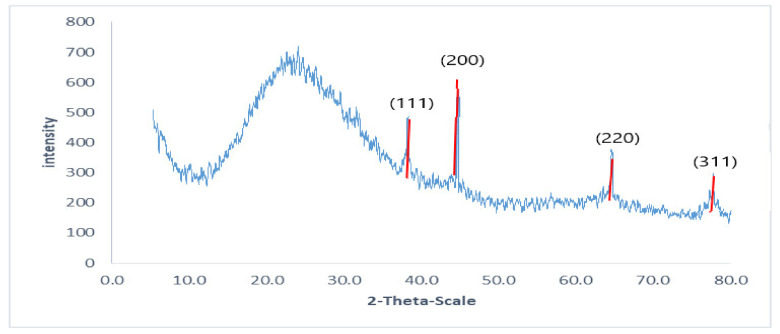
X-ray diffraction pattern of the biosynthesized AgNPs using GTLE.

The intense peaks were noticed at the 2θ scale of 38.26, 44.47, 64.71, and 77.73 corresponding to the (111), (200), (220), and (311) planes for silver, respectively (Figure 6).

#### 2.2.7. Scanning Electron Microscope (SEM)

**Figure 7:**
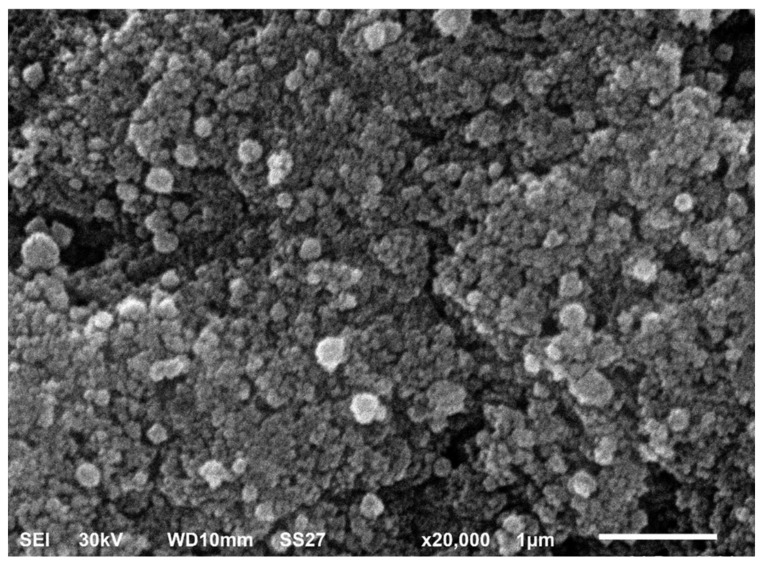
SEM of AgNPs biosynthesized using GTLE.

SEM is a useful tool for investigating an object’s surface images. It can precisely illustrate the particle size, shape, and distribution of the tested material. In addition, it can determine the morphological appearance of the studied object and determine whether its size is at the micro- or nanoscale. SEM analysis of the biosynthesized AgNPs revealed that they are spherical in shape with a tendency to aggregate (Figure 7).

### 2.3. Total Content of Flavonoids and Polyphenolics

Total flavonoids were found to have a content of 162.98 mg/g equivalent to quercetin, while total polyphenols had a content of 287.89 mg/g equivalent to gallic acid. Findings indicate that *G. thailandica* possesses high contents of polyphenols and flavonoids.

### 2.4. Antioxidant Activity

The antioxidant activity of GTLE was investigated in this study using radical scavenging and metal-reducing assays. Radical scavenging assays used were 2,2-diphenyl-1-picrylhydrazyl (DPPH) and 2,2’-azino-bis(3-ethylbenzothiazoline-6-sulfonic acid) (ABTS) tests. GTLE exhibited antioxidant activity by DPPH and ABTS as (IC_50_ 72.91 µg/mL) and (211.60 mg Trolox equivalents (TE)/mg), respectively. The metal-reducing assay used was the ferric reducing antioxidant power assay (FRAP) and the activity of GTLE was 70.95 mg TE/mg.

### 2.5. In Vitro Antibacterial Activity

#### 2.5.1. In Vitro Susceptibility Testing

The green-synthesized AgNPs exhibited antibacterial activity against *S. aureus* clinical isolates, as they resulted in clear zones around the AgNPs discs by disc diffusion method. The broth microdilution method was utilized to identify the MIC values of AgNPs, and they ranged from 4 to 64 µg/mL. All the following tests were carried out after treatment of the tested isolates with 0.5 MIC values.

#### 2.5.2. Time Kill Curve

**Figure 8:**
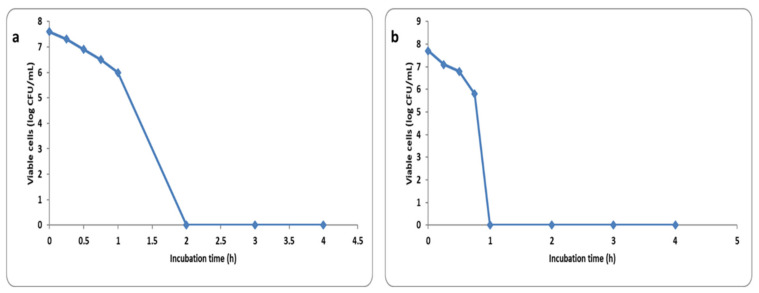
Time kill plot of (**a**) 4× MIC and (**b**) 8× MIC of the green synthesized AgNPs against *S. aureus* isolates.

The number of colony-forming units (CFU) per milliliter was reduced by more than 3 log units after incubation of *S. aureus* cells for 2 h with 4× MIC and 1 h with 8× MIC in 58.34% and 47.92% of the isolates, respectively. A representative example for the reduction of the CFU/mL is shown in Figure 8.

#### 2.5.3. Membrane Integrity and Permeability

**Figure 9:**
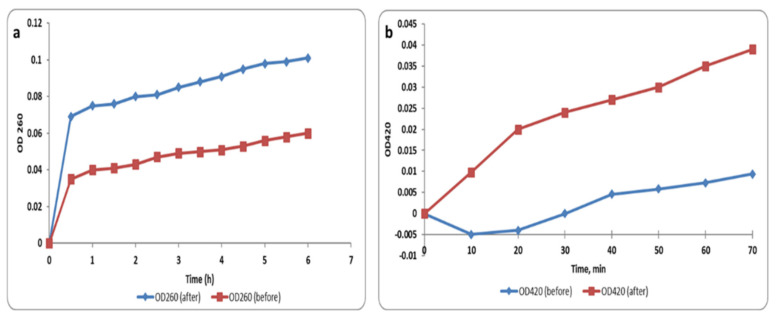
Line chart showing (**a**) the cell membrane integrity and (**b**) the membrane permeability of a representative *S. aureus* isolate before and after treatment with the green-synthesized AgNPs (at concentrations equal to 0.5 MIC values).

We investigated the cell membrane integrity of *S. aureus* isolates after treatment with the green synthesized AgNPs (at concentrations equal to 0.5 MIC values) via detection of the release of the materials (DNA and RNA), which absorb at 260 nm, from the bacterial isolates. Herein, we found that the membrane integrity significantly decreased (*p* < 0.05) in 45.8% of the isolates after treatment with AgNPs. Figure 9a illustrates a representative example.

When the bacterial membrane permeability increases, *O*-nitrophenyl-β-galactopyranoside (ONPG) enters the bacterial cytoplasm in a large amount. In the cytoplasm, ONPG is broken down into *O*-nitrophenol (ONP) by a β-galactosidase enzyme that is present in the cytoplasm. Thus, the membrane permeability was tracked by monitoring the absorbance at OD_420_ (the yellow color of ONP can absorb at 420 nm) with time. The membrane permeability significantly increased (*p* < 0.05) in 56.25% of *S. aureus* isolates after treatment with AgNPs and an illustrative example is revealed in Figure 9b.

#### 2.5.4. Membrane Depolarization

**Figure 10:**
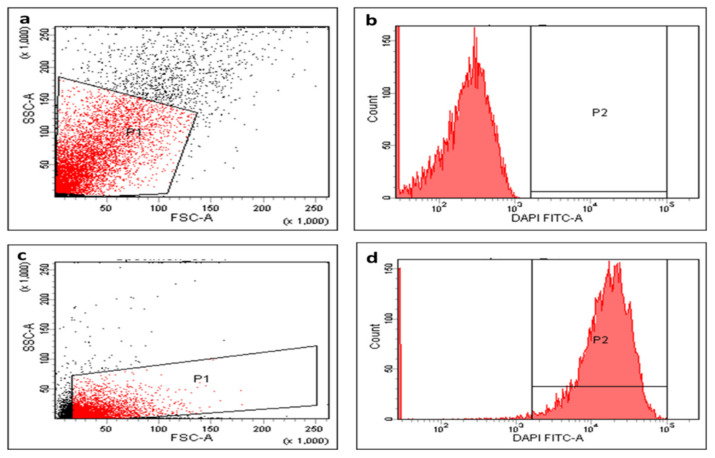
Flowcytometric chart (**a**) dot plot, (**b**) histogram (fluorescent gap = 67.1%) before treatment, (**c**) dot plot, and (**d**) histogram (fluorescent gap = 35.1%) after treatment of a representative *S. aureus* isolate with the green-synthesized AgNPs.

Membrane depolarization was determined in the tested isolates using DiBAC4(3) (bis-(1,3-dibutylbarbituric acid) trimethine oxonol) fluorescent stain. This is a membrane potential-sensitive stain that can enter the depolarized cell cytoplasm and bind to the intracellular proteins exhibiting an enhanced fluorescence. In the current investigation, we noticed that treatment with AgNPs exhibited a considerable reduction (*p* < 0.05) in the membrane potential in 35.42% of *S. aureus* isolates. A demonstrative example of the decrease in the membrane potential after AgNPs treatment is presented in Figure 10.

#### 2.5.5. SEM Examination

**Figure 11:**
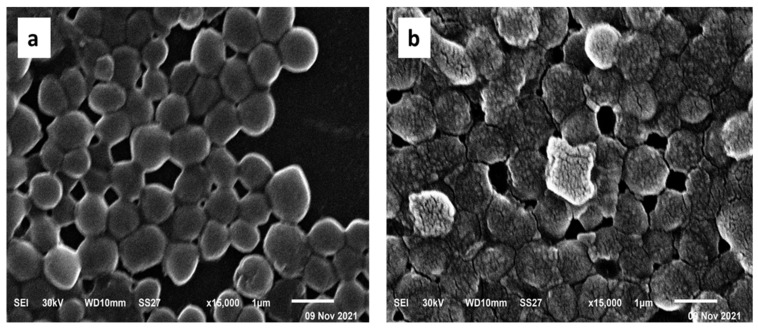
Scanning electron micrograph of a representative *S. aureus* isolate (**a**) before treatment and (**b**) after treatment with the green-synthesized AgNPs.

The ultrastructural and morphological changes of *S. aureus* cells treated with the green-synthesized AgNPs were observed by SEM (Figure 11). The electron micrographs obtained by SEM revealed that the untreated cells had sphere-shaped, intact, smooth surfaces. On the other hand, the treated cells had a deformed and distorted shape.

#### 2.5.6. Efflux Activity

The efflux activity of the tested *S. aureus* isolates was assessed by testing the capability of the cells to pump out ethidium bromide (EtBr) to the surrounding medium by the EtBr cartwheel method. Herein, we categorized the efflux activity of *S. aureus* isolates into three classes; negative, intermediate, and positive efflux activity as presented in Table 2. Eleven (22.92%) *S. aureus* isolates exhibited a reduction in their efflux activity when treated with the green-synthesized AgNPs. The efflux activity of these isolates changed from positive to either intermediate or negative.

#### 2.5.7. Quantitative Real-Time PCR (qRT-PCR)

**Figure 12:**
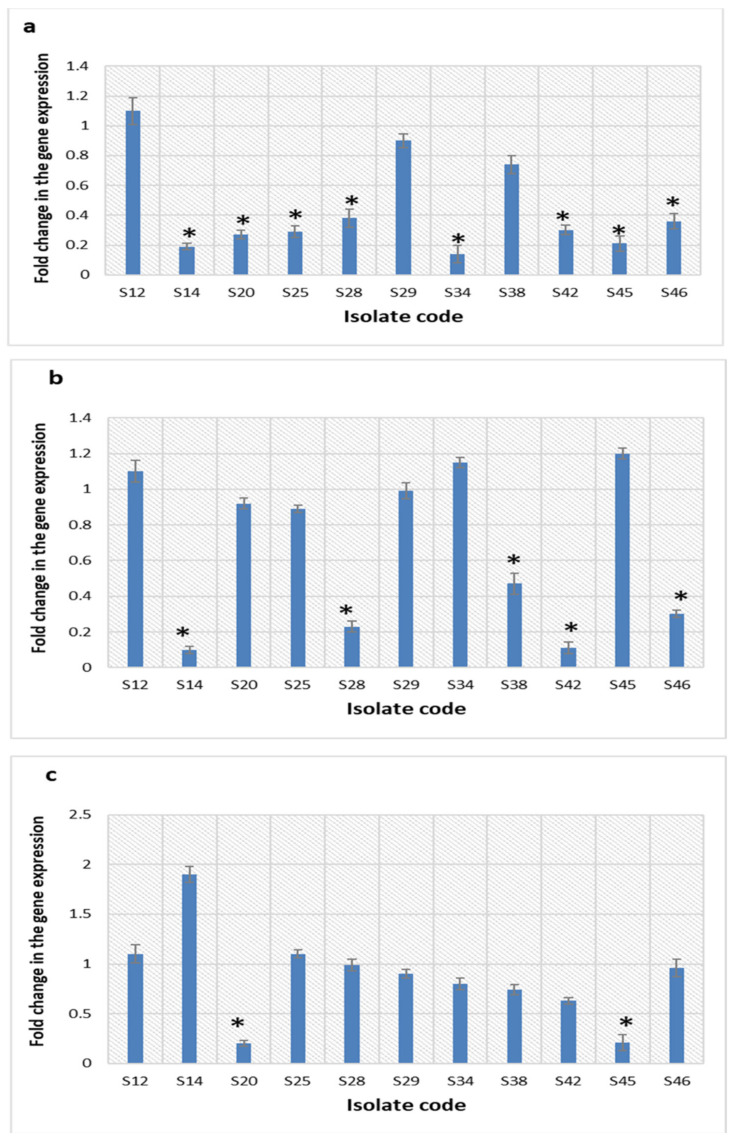
Bar charts showing the transcriptional level fold changes of (**a**) *nor*A, (**b**) *nor*B, and (**c**) *nor*C genes after treatment with the green-synthesized AgNPs. * represents a significant decrease in the fold change.

qRT-PCR was utilized for more in-depth exploration of the impact of the green-synthesized AgNPs on the efflux pump activity of the tested isolates. *S. aureus* bacteria (*n* = 11) that displayed a decline in their efflux pump activity by the EtBr cartwheel method were selected for this assay. We found that the transcriptional levels of *nor*A, *nor*B, and *nor*C efflux pump genes decreased in 72.73%, 45.45%, and 18.18% of the tested isolates, respectively, after treatment with AgNPs. The fold changes mean values of *nor*A, *nor*B, and *nor*C efflux pump genes ranged from 0.11 to 0.47, respectively, as presented in Figure 12.

### 2.6. In Vivo Antibacterial Activity

The impact of AgNPs was investigated on macroscopic healing and skin histology following excisional wound healing as follows.

#### 2.6.1. Macroscopic Healing

**Figure 13:**
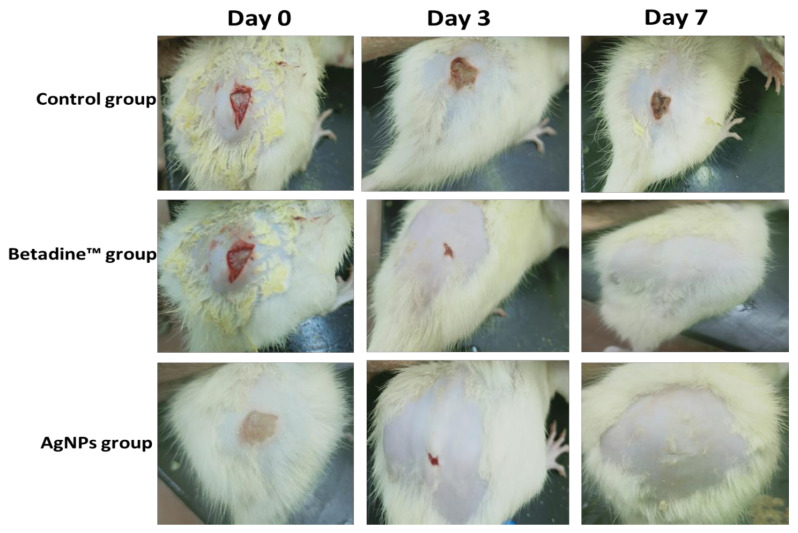
Macroscopic examination of the wound healing of the control, Betadine™, and AgNPs rat groups over the days 0, 3, and 7 starting from the day of wound creation (day 0).

The rates of the macroscopic wound healing of the studied groups were inspected on days 0, 3, and 7, considering the day of the wound creation as day 0. Betadine™ and AgNPs groups demonstrated full and notable wound healing in comparison with the control group (Figure 13).

**Figure 14:**
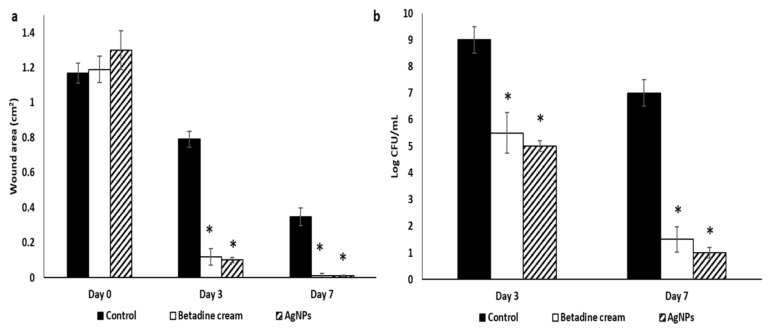
Wound characters on days 0, 3, and 7 in the different rat groups including (**a**) wound area, and (**b**) quantification of CFU/mL. * represents a significant decrease (*p* < 0.05).

Betadine™ and AgNPs groups exhibited significant wound healing on day 3 with wound healing percentages of 90.19 and 92.3, respectively, compared to the control group. Furthermore, they exhibited full wound healing on day 7 (with wound healing percentages of 99.02 and 99.23, respectively, in comparison with the control group (Figure 14a).

In addition, both Betadine™ and AgNPs groups exhibited a significant decrease in CFU/mL in comparison with the control group (Figure 14b). 

#### 2.6.2. Histological Examination

**Figure 15:**
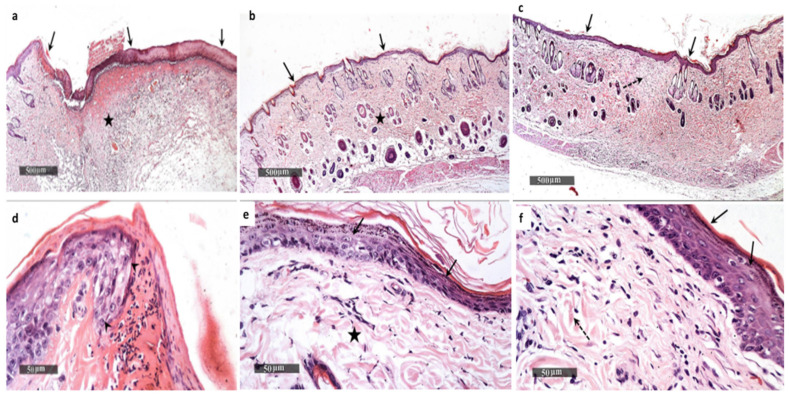
Histological examination of the wounds of rats on the seventh day. The control group (**a**,**d**) showed the presence of a wide area of epidermal loss in addition to ulceration in the wound gap (black arrow) with adjacent thickening in the epidermis (arrowhead). There was a highly cellular granulation tissue that is rich in inflammatory cells (black star). The Betadine™ group (**b**,**e**) showed an intact thin epidermal layer with intact keratinocytes and subcellular details (arrow). There was an intact layer of the dermis with a normal distribution of cellular elements and abundant well-organized collagen fibers (black star). The AgNPs group (**c**,**f**) showed efficient wound healing with complete epidermal re-epithelialization as well as wound closure (black arrow). There was more abundant fibroblastic activity with dermal granulation tissue rich in collagen (dashed arrow).

The skin section of the wounds of the control group showed a wide area of epidermal loss and infiltration with inflammatory cells with granulation tissue formation (Figure 15a,d).

On the other hand, the skin section of the wounds of the Betadine™ group showed enhanced epidermal re-epithelialization and wound closure with more abundant fibroblastic activity and more collagen-rich dermal granulation tissue (Figure 15b,e). The section of the AgNPs treated group also exhibited complete wound healing with continuous epidermis and underlying fibrosis and collagenosis (Figure 15c,f).

## 3. Discussion

The widespread community and hospital-acquired infections caused by *S. aureus* isolates are a global consideration. In addition, these pathogenic bacteria are largely related to resistance to many commercially available antimicrobial agents [17]. Thus, many researchers have focused their studies on the exploration of new antimicrobial compounds against various types of pathogenic bacteria such as *S. aureus*. Natural products such as plants are showing promising antimicrobial activity with relatively low toxicity, low cost, and high bioavailability [18,19,20,21,22,23]. Herein, we used GTLE for the green synthesis of AgNPs. AgNPs drew our attention owing to their documented versatile activities in the literature, such as their antimicrobial and anti-inflammatory properties in addition to their wound healing promotion capability. AgNPs are currently utilized as interesting tools to face many emerging therapeutic challenges [24]. Despite their advantageous properties, the synthesis of AgNPs can be a high-cost process and a harmful approach. This is because of the utilization of chemical compounds and the possible production of certain harmful by-products [25,26,27]. Therefore, we decided to rely on the green synthesis of AgNPs to avoid these drawbacks. Many natural products such as plant extracts can be used in the green biosynthesis of AgNPs. In this case, the bioactive compounds of plants reduce silver to form AgNPs. In this way, we avoid the use of chemical reducers with their accompanying problems. The fundamental principle of the green methods is to utilize nontoxic biomolecules for the synthesis of nanoparticles via the reduction of metal ions in an aqueous solution. Most biomolecules such as DNA, proteins, and enzymes are quite expensive, easily decomposed, and vulnerable to being contaminated. On the other hand, many plant extracts are available, affordable, and stable against most environmental conditions (such as pH, temperature, and salt concentration) [5,28,29].

Nontoxic, environmentally friendly methods were used to synthesize bioinspired silver nanoparticles from GTLE. The quantification of the flavonoids and phenolic acids of *G. thailandica* was evaluated by the HPLC-DAD technique using 20 standard compounds. A few studies have documented the presence of these types of bioactive compounds in different *Gardenia* species. The HPLC-DAD analysis detected major phenolic compounds that are reported to possess antitumor, antibacterial, antioxidant, antidiabetic, and antihypercholesterolemic activities through different pathways [13]. Six phenolic acids were recognized (chlorogenic, rosmarinic, cinnamic, vanillic, *p*-coumaric, and syringic acid). In addition, four flavonoids were identified (quercetin-3-rutinoside, apigenin-7-glucoside, luteolin, and chrysin). The results of the HPLC-DAD analysis of *G. thailandica* revealed the presence of quercetin-3-rutinoside at a concentration of 2477.37 μg/g as the major flavonoid glycoside, while chlorogenic acid was the major phenolic acid in the extract at a concentration of 1441.03 μg/g, followed by rosmarinic acid at a concentration of 796.67 μg/g.

The antibacterial activity of the green-synthesized AgNPs was investigated both in vitro and in vivo. AgNPs exhibited antibacterial activity against *S. aureus* clinical isolates with MIC values that ranged from 4 to 64 µg/mL. Many studies have recorded that green-synthesized AgNPs have antibacterial activity against different pathogenic bacteria [30,31,32,33]. The short reproductive time of *S. aureus* bacteria is one of the principal reasons for the infectivity of such pathogenic bacteria [30]. Therefore, we investigated the impact of AgNPs on the time-kill curve of the tested *S. aureus* isolates and we found that the CFU/mL of *S. aureus* isolates was reduced by more than 3 log units after its incubation for 2 h with 4 × MIC and 1 h with 8 × MIC in 58.34% and 47.92% of the isolates, respectively. 

As the bacterial cell membrane is an important target for several antimicrobials, we investigated the impact of the green-synthesized AgNPs on membrane characteristics including the membrane integrity, permeability, and depolarization. The bacterial cell membrane is considered to be a barrier with a selective permeability character, and the loss of this property can lead to cell death [34]. Herein, we investigated the membrane integrity of the tested bacteria before and after treatment with AgNPs by observing the leakage of materials absorbing 260 nm over time. We observed that treatment with AgNPs resulted in a massive reduction (*p* < 0.05) in the membrane integrity in 45.8% of the isolates. Many different techniques can be used for the evaluation of membrane permeability. In the current study, we used the ONPG method, which relies on the concept that when the bacteria are losing the ability to control their membrane permeability, the penetration of this compound increases [34]. Our results showed that the membrane permeability of the tested bacterial cells significantly increased (*p* < 0.05) in 56.25% of the isolates after treatment with AgNPs. Owing to the importance of the membrane potential in bacterial viability, we used DiBAC4, a fluorescent probe that enters the cell and links to the intracellular proteins when the membrane potential is lost. Here, the green-synthesized AgNPs resulted in a considerable reduction (*p* < 0.05) in membrane potential in 35.42% of *S. aureus* isolates.

SEM is widely utilized in microbiological research to study the different changes that occur in the ultrastructure and morphology of the bacterial cells when they are treated with antimicrobial agents [35]. Consequently, we used SEM in this study to explore the cell surface characters and external cell morphology to gain the benefit of the higher resolution of SEM when compared to light microscopes. Herein, we noticed that the AgNP-treated bacterial cells had a deformed and distorted shape in comparison with the non-treated ones.

The function of efflux pump proteins is to transfer harmful substances out of bacterial cells [26]. Therefore, efflux pumps are an important resistance mechanism to many antibiotics. In the current study, 22.92% of *S. aureus* isolates presented a reduction in their efflux activity after treatment with AgNPs. Efflux pumps in *S. aureus* bacteria are encoded by *nor*A, *nor*B, and *nor*C genes. For further elucidation of the effect of AgNPs on the efflux activity of the 11 *S. aureus* isolates that displayed a decline in their efflux activity by EtBr cartwheel assay, qRT-PCR was utilized. We noticed that treatment with AgNPs resulted in a substantial decrease in the expression of *nor*A, *nor*B, and *nor*C genes in 72.73%, 45.45%, and 18.18% of the tested isolates, respectively. Generally, metal nanoparticles could inhibit the efflux pump activity of bacteria by two mechanisms. The first possible mechanism is by direct binding to the efflux pumps’ active site and the second mechanism is by disturbing the efflux kinetics [36]. 

The process of wound healing is associated with certain biological events such as re-epithelialization, fibroplasia in addition to extracellular matrix production. Many natural agents were found to produce satisfactory results in wound healing when compared to the chemical compounds, with the advantages of low cost and low toxicity [37]. Consequently, we used GTLE to synthesize AgNPs and investigated their effect on wound healing in rats with wounds infected with *S. aureus* isolates after seven days of treatment. The group treated with AgNPs exhibited notable wound healing when compared to the other groups. On the histological level, the AgNP-treated group displayed accelerated wound healing with complete epidermal re-epithelialization, abundant fibroblastic activity, formation of collagen-rich dermal granulation tissue, and minimal infiltration of inflammatory cells. 

## 4. Materials and Methods

### 4.1. Plant Materials and Extract Preparation

*Gardenia thailandica* Tirveng. leaves were collected from a private garden on the Egypt Alexandria desert road. Esraa Ammar (Plant Ecology, Botany Department, Faculty of Science, Tanta University) confirmed the plant’s identification. A voucher sample (PGA-GT-128-W) was maintained in the Tanta University Department of Pharmacognosy’s herbarium. The powdered plant (650 g) was extracted with methanol using a maceration method (3 × 5 L). The extract was concentrated using a rotary evaporator to obtain a residue (7.89 g).

### 4.2. Drugs and Chemicals

All the chemicals and solvents used in this study were bought from Sigma-Aldrich (St. Louis, MO, USA) and were of high analytical quality.

### 4.3. HPLC-DAD of GTLE

An autosampler and a diode-array detector are included in the Agilent Technologies 1100 series liquid chromatography. 

The analytical column was an Eclipse XDB-C18 (150 × 4.6 µm; 5 µm) with a C18 guard column (Phenomenex, Torrance, CA, USA). Acetonitrile (solvent A) and 2% acetic acid in water (Solvent B) made up the mobile phase. The flow rate was held constant at 0.8 mL/min for a total run of 70 min, and the gradient program was as follows: 100% B to 85% B in 30 min, 85% B to 50% B in 20 min, 50% B to 0% B in 5 min, and 0% B to 100% B in 5 min. The injection volume was 50 µL, and peaks for benzoic acid, cinnamic acid derivatives, and flavonoids were found simultaneously at 280, 320, and 360 nm, respectively. All samples were filtered using a 0.45 µm Acrodisc syringe filter (Gelman Laboratory, Michigan, USA) before injection. The peaks were identified using congruent retention durations and UV spectra, which were then compared to the standards.

### 4.4. Green Synthesis of AgNPs

One millimolar of an aqueous solution of silver nitrate (AgNO_3_) was prepared and maintained in a cool dark area. For reduction of Ag+ ions, 10 mL of GTLE was added separately into 90 mL of an aqueous solution of 1 mM AgNO_3_ and incubated overnight at room temperature in a dark area. The development of AgNPs was indicated by the production of a yellowish-brown color. The produced solutions were directly subjected to TEM and UV measurements. Centrifugation at 4000 rpm for 30 min was followed by a series of washing in distilled water and filtration to obtain pure AgNPs. The pure AgNPs were further characterized by FTIR, HR-TEM, XRD, zeta potential, and SEM [38,39,40,41]. 

### 4.5. Characterization of AgNPs

#### 4.5.1. UV-Vis Spectroscopy

UV-Vis spectroscopy of the green-synthesized AgNPs was monitored using a UV–Vis spectrophotometer (Shimadzu, Kyoto, Japan) after dilution with distilled water.

#### 4.5.2. FTIR

The different functional groups of the produced AgNPs were measured by FTIR spectrometer (Jasco, Tokyo, Japan) in the range of 4000–400 cm^−1^.

#### 4.5.3. HR-TEM

The morphology of the particles (shape and dimensions) in addition to SAED were examined by TEM. (JEOL-JEM-1011, Kyoto, Japan) and HR-TEM at 200 kV (JEOL-JEM-2100, Kyoto, Japan). Three milliliters of the sample were placed on the copper grid for TEM and HR-TEM examination and allowed to dry at room temperature for 15 min.

#### 4.5.4. Zeta Potential and DLS

Particle size, homogeneity of distribution, and zeta potentials of AgNPs were examined using a zeta sizer nano ZN (Malvern Panalytical Ltd., England, UK). Before the measurements, an aliquot of nanoparticles was diluted with ultra-purified water and then sonicated for 15 min.

#### 4.5.5. XRD

The XRD analysis was performed as a surface chemical analysis tool for the characterization of metal nanoparticles [42]. An XPERT-PRO-PANalytical Powder Diffractometer (PAN-alytical B.V., Almelo, The Netherlands) was used to perform XRD utilizing a monochromatic radiation source Cu-K α radiation (θ = 1.5406 Å) at 45 kV and 30 mA at ambient temperature. The silver nano-powder intensity data were gathered over a 2θ range of 4.01°–79.99°.

#### 4.5.6. SEM

The morphology of the biosynthesized AgNPs was observed using SEM (TM1000, Hitachi, Chiyoda, Japan) as described previously [43].

### 4.6. Determination of the Total Content of Flavonoids and Polyphenols

The total flavonoid concentration was determined by colorimetric analysis of serial dilutions of the extract using the aluminum chloride technique and quercetin as a reference [44]. Using the Folin–Ciocalteu technique and gallic acid as a reference, the total content of polyphenols was determined [45]. The measured contents were expressed as mg/g equivalent of the corresponding standard for each method.

### 4.7. Antioxidant Activity of GTLE

#### 4.7.1. The DPPH Radical Scavenging Capacity

The DPPH radical scavenging capacity of GTLE was evaluated according to the method of Boly et al. [46]. The decrease in DPPH color intensity was measured at 540 nm using the following equation:

$$\begin{array}{c} Percentage\;inhibition=\\ =\frac{\left(\mathrm{Average}\;\mathrm{absorbance}\;\mathrm{of}\;\mathrm{blank}-\mathrm{average}\;\mathrm{absorbance}\;\mathrm{of}\;\mathrm{the}\;\mathrm{test}\right)\;}{\mathrm{Average}\;\mathrm{absorbance}\;\mathrm{of}\;\mathrm{blank}}\times 100 \end{array}$$

The value of IC_50_ was calculated as previously described [47].

#### 4.7.2. The ABTS Radical Scavenging Capacity

The assay was performed as previously reported [48]. Using the linear regression equation taken from the calibration curve, the results are presented as µM Trolox equivalents (TE)/ mg samples (linear dose-inhibition curve of Trolox).

#### 4.7.3. FRAP Assay

The ferric reducing ability assay was conducted according to the method of Benzi et al. [49]. The result is expressed as µM TE/mg sample using the linear regression equation derived from the calibration curve (linear dose-response curve of Trolox).

### 4.8. In Vitro Antibacterial Activity

#### 4.8.1. Bacterial Isolates

A total of 48 *S. aureus* clinical isolates were acquired from Tanta University Hospital. *S. aureus* isolates were microscopically examined and were biochemically identified as previously described [50]. *Staphylococcus aureus* (ATCC 29231) was utilized as a reference strain.

#### 4.8.2. Susceptibility Testing

##### Disk Diffusion Method

The antimicrobial activity of AgNPs against *S. aureus* clinical isolates was performed using the Kirby–Bauer disk diffusion method [51]. Mueller–Hinton agar (MHA) (Merck, Germany) plates were inoculated with the bacterial isolates using sterile swabs. Sterile discs were thoroughly saturated with vancomycin and sterile water as positive and negative controls, respectively. In addition, a third disc was saturated with the green-synthesized AgNPs was added. Then, the disks were located on the MHA plates and incubated for 24 h at 37 °C. The formed inhibition zones were observed indicating antibacterial activity.

##### Minimum Inhibitory Concentration (MIC) Determination

The MIC values of the green-synthesized AgNPs were determined in a 96-well microdilution plate using the broth microdilution method [52]. The green-synthesized AgNP solution (500 µg/mL) was twofold diluted with each bacterial inoculum in 100 µL of MHB (10^6^ CFU/mL). Each microtitration plate had a negative control (MHB only) and positive control (MHB containing bacteria). Each well of the microtitration plate was loaded with 30 µL of the resazurin solution and then the plates were incubated at 37 °C for 24 h. The variations in the color were detected.

#### 4.8.3. Time Kill Curve

This was performed as previously reported [53]. Briefly, the green-synthesized AgNPs solution was diluted by MHB containing the bacterial suspensions to obtain a final concentration of 0× MIC, 0.5× MIC, 1× MIC, 2× MIC, 4× MIC, and 8× MIC for each bacterial isolate. The obtained cultures were then incubated in a shaking incubator at 37 °C. Aliquots of the cultures (100 µL) were distributed on the surface of MHA plates at 0, 0.25, 0.5, 1, 2, and 4 h. After incubation at 37 °C for 24 h, the colonies detected on the MHA plates were quantified in CFU/mL.

#### 4.8.4. Membrane Integrity and Permeability

##### Membrane Integrity Assay

The effect of the green-synthesized AgNPs on the integrity of the cell membrane of the tested isolates was studied by monitoring the release of materials that have absorbance at 260 nm (A260) [54]. In brief, the optical density (OD) of the overnight bacterial cultures in nutrient broth was adjusted to be 0.4 at 630 nm. Then, the bacterial suspensions were centrifuged at 11,000× *g* for 10 min and the obtained pellets were resuspended in 0.5% NaCl solution and their absorbance was adjusted to 0.7 at 420 nm. The membrane integrity was assessed by checking the discharge of materials that have absorbance at 260 nm from the bacterial cytoplasm to the surrounding media over time using a UV/Vis spectrophotometer (SHIMADZU, Kyoto, Japan).

##### Membrane Permeability Assay

Membrane permeability was explored by quantifying the exit of a β-galactosidase enzyme from the bacterial cytoplasm using the substrate of the enzyme (ONPG) [55]. Briefly, 2% lactose was added to the overnight bacterial suspension in nutrient broth. This mixture was then centrifuged, and the obtained pellet was thoroughly rinsed using phosphate-buffered saline (PBS) and resuspended in NaCl solution (0.5%). Finally, each bacterial suspension (1.6 mL) was supplemented with 150 µL of ONPG solution (34 mM). The produced ONP was detected over time using an ELISA reader (Sunrise Tecan, Männedorf, Switzerland) to monitor the increase in absorbance at 420.

#### 4.8.5. Membrane Depolarization

This test was carried out using DiBAC4(3), a fluorescent stain used for staining the tested bacterial cells (both treated and untreated with the green-synthesized AgNPs) [56]. A FACSVerse flow cytometer (BD Biosciences, Franklin Lakes, New Jersey, USA) was used to analyze the staining of the cells.

#### 4.8.6. SEM

The morphological changes of the AgNPs treated *S. aureus* isolates in comparison with the non-treated ones were inspected by SEM (Hitachi, Chiyoda, Japan) as described by McDowell and Trump [57].

#### 4.8.7. Efflux Activity

Efflux activity was tested by the EtBr cartwheel method [58] before and after treatment with AgNPs (at 0.5 MIC values) using the reference strain as a negative control. In brief, bacterial suspensions were inoculated as redial lines onto tryptic soy agar (TSA) plates using swabs and were incubated at 37 °C for 18 h. The TSA plates were supplied with EtBr (with concentrations that ranged from 0.5 to 2.5 mg/L). After incubation, the lowest EtBr concentrations that led to fluorescence production by the bacterial isolates were recorded by UV-Vis spectrophotometer (SHIMADZU, Kyoto, Japan). *S. aureus* isolates were then classified according to the recorded EtBr minimum concentrations as follows: isolates with no efflux activity are those that emitted fluorescence at an EtBr concentration of 0.5 mg/L, isolates with intermediate efflux activity are that emitted fluorescence at an EtBr concentration of 1–2.0 mg/L, and isolates with positive efflux activity are those that emitted fluorescence at an EtBr concentration of 2.5 mg/L.

#### 4.8.8. qRT-PCR

We used qRT-PCR for detection of the expression levels of the genes encoding efflux pumps (*nor*A, *nor*B, and *nor*C) in *S. aureus* isolates after treatment with AgNPs. In brief, total RNA was extracted from the pellets of overnight cultures of *S. aureus* isolates using the Purelink™ RNA Mini Kit (Thermo Scientific, Waltham, USA) according to the instructions of the manufacturer. The extracted RNA was then converted into cDNA by power™ cDNA synthesis kit (iNtRON Biotechnology, Seoul, Korea) as described by the manufacturer. Rotor-Gene Q 5plex (Qiagen, Hilden, Germany) was used for performing qRT-PCR for the calculation of the efflux pump gene expression fold changes. The sequences of the utilized primers in addition to the sequence of the housekeeping gene (16S rRNA) primer are presented in Table S1 [59,60]. The levels of the relative expression of the tested genes were quantified by the 2^−ΔΔCt^ method, considering the gene expression levels in the isolates before treatment to be 1 [61]. The statistically significant fold changes were those with two or more-fold changes (either increasing or decreasing) [62].

### 4.9. In Vivo Antibacterial Activity

#### 4.9.1. Animals

We obtained 30 white albino male rats (190–210 g and 8 weeks old) from the animal house at the Faculty of Veterinary Medicine, Cairo University. All rats were kept in an environment with a constant temperature of 25 ± 2 °C and a pathogen-free atmosphere with a 12-h light/dark cycle. They were permitted free access to a standard pellet diet in addition to filtered water. The rats were given one week for acclimatization before being utilized in the research. The in vivo experiment performed in the current study followed the standards of the use of the laboratory animals authorized by the Faculty of Pharmacy Research Ethical Committee (Tanta University, Al Gharbiyah, Egypt) with approval number TP/RE/11-21-P-001.

#### 4.9.2. Wound Model

The 30 rats were distributed into three groups, randomly, each with 10 rats. The groups were as follows: the control group (0.9% normal saline), Betadine™ ointment (Mundi pharma as standard drug) group, and AgNP group. The rats were anesthetized using diethyl ether; then a small area was carefully shaved on their backs. After that, full-thickness excisional wounds were created and infected with *S. aureus* bacteria (10^6^ CFU/mL). For 7 days, the groups were administered the drugs topically on the surface of the wound [63].

#### 4.9.3. Macroscopic Wound Healing

The day of the wound creation was considered as day 0, and the wound healing process was observed for 7 days starting from day 0. The wound images were taken on days 0, 3, and 7 using a digital camera. Wound areas (cm^2^) were calculated on days 0, 3, and 7 for evaluation of the healing efficacy using a ruler beside the wounds [64]. The percentage of the wound healing was calculated using the following equation [65]:

$$Percentage\;of\;wound\;healing=\frac{wound\;area\;at\;day\;0-wound\;area\;at\;n\;th\;day}{wound\;area\;at\;day\;0}\times 100$$

where *n* represents day 3 and day 7.

In addition, on days 3 and 7 post-wounding, wound tissues were excised and homogenized in PBS (10 mL), 10-fold serially diluted in MHB, and plated onto mannitol salt agar plates for CFU quantification after overnight incubation at 37 °C [66,67].

#### 4.9.4. Histological Examination

The entire wound was isolated for histological examination at the end of the experiment, with a margin of about 5 mm of the surrounding intact skin. The skin sections were fixed using 10% formalin solution (pH 7.4) overnight. Then, they were processed using a series of alcohol and xylene grades. Following that, the tissues were inserted in paraffin wax at 65 °C. The blocks of tissues were cut into sections of 5 μm thickness, stained with hematoxylin and eosin (H&E), and finally viewed using a light microscope [63].

### 4.10. Statistical Analysis

All tests were accomplished in triplicate and the obtained results are presented as mean ± standard deviation (SD). Prism 8^®^ software (GraphPad, Inc., San Diego, CA, USA) was utilized to assess the statistical significance at *p* < 0.05.

## 5. Conclusions

According to our results, GTLE includes phenolic compounds. These substances have the ability to form and stabilize AgNPs. In conclusion, the green-synthesized AgNPs by GTLE exhibited good antibacterial activity both in vitro and in vivo against *S. aureus* clinical isolates. They significantly decreased (*p* < 0.05) the membrane integrity of 45.8% of the isolates. In addition, they reduced the membrane potential of 35.42% of isolates by flow cytometry. AgNPs also resulted in morphological and ultrastructural changes in the tested isolates, as revealed by SEM. Furthermore, they resulted in a significant reduction in efflux activity in 41.67% of the tested isolates. For more in-depth study of the impact of AgNPs on efflux activity, qRT-PCR was utilized to examine the relative gene expression of the efflux pump genes (*nor*A, *nor*B, and *nor*C). The in vivo examination was performed on wounds infected with *S. aureus* bacteria in rats. The group treated with AgNPs was characterized by epidermis regeneration and reduction in the infiltration of the inflammatory cells. Therefore, the green-synthesized AgNPs by GTLE could be a future alternative to chemical compounds and antimicrobials that are currently used to induce healing of wounds, especially infected ones. 

## Figures and Tables

**Table 1:** Chemical composition analysis of the phenolic and flavonoid compounds of GTLE by HPLC-DAD.

No	Retention Time (RT)	Compound	Concentration (μg/g) *
1	3.93	Gallic acid	ND
2	6.61	Protocatechuic acid	ND
3	9.91	*p*-hydroxybenzoic acid	ND
4	11.44	Gentisic acid	ND
5	12.18	Cateachin	ND
6	12.41	Chlorogenic acid	1441.03
7	13.33	Caffeic acid	ND
8	16.18	Syringic acid	10.09
9	17.69	Vanillic acid	44.17
10	20.26	Ferulic acid	ND
11	21.03	Sinapic acid	ND
12	22.26	*p*-coumaric	26.16
13	22.97	Quercetin-3-Rutinoside	2477.37
14	27.43	Rosmarinic acid	796.67
15	28.71	Apigenin-7-glucoside	605.60
16	30.04	Cinnamic acid	436.06
17	34.79	luteolin	753.18
18	39.50	Apigenin	ND
19	53.34	Kaempferol	ND
20	58.42	Chrysin	152.71

* ND stands for none detected.

**Table 2:** Efflux activity of *S. aureus* isolates determined using EtBr cartwheel method, before and after treatment with AgNPs.

EtBr Conc. (mg/L) *	Number of Isolates (Before Treatment)	Number of Isolates (After Treatment)
≤0.5	5	7
1	10	12
1.5	11	13
2	8	13
2.5	14	3

* Concentration of EtBr at which *S. aureus* bacteria started to produce fluorescence. The isolates which emit fluorescence at 0.5 mg/L, 1–2 mg/L, and 2.5 mg/L lack efflux activity, have intermediate efflux activity, and have positive efflux activity, respectively.

## Data Availability

Data is contained within the article and supplementary materials.

## Supplementary Materials

The following supporting information can be downloaded at: https://www.mdpi.com/article/10.3390/ph15020194/s1, Table S1. The sequences of the primers used in qRT-PCR.
