# Supplementary material for: In Vivo and In Vitro Antimicrobial Activity of Biogenic Silver Nanoparticles against Staphylococcus aureus Clinical Isolates
Source: Pharmaceuticals (Basel). 2022 Feb 3;15(2):194. doi: 10.3390/ph15020194 (PMC8878289; doi:10.3390/ph15020194)
Supplement: Supplementary file 1 [file pharmaceuticals-15-00194-s001.zip › pharmaceuticals-1567152-supplementary.pdf]

Supplementary data

Table S1. The sequences of the primers used in qRT-PCR.

Gene	Primer	Sequence
*nor*A	Forward	5′-GACATTTCACCAAGCCATCAA-3′
	Reverse	5′-TGCCATAAATCCACCAATCC-3′
*nor*B	Forward	5′-GCTACACCATCAACAGATACAGCAA-3′
	Reverse	5′-ACTCAATGCGACGCCAAA-3′
*nor*C	Forward	5′-TGGGTTGGAGATGGATTTTC-3′
	Reverse	5′-ACAATTAGCCCTGCAACGTC-3′
16srRNA	Forward	5′-CGTGGAGGGTCATTGGA-3′
	Reverse	5′-CGTTTACGGCGTGGACT-3′
